# Unusual MRI Findings in a Polio Survivor

**DOI:** 10.1155/2016/3179621

**Published:** 2016-03-16

**Authors:** Masaaki Sakamoto, Hitoshi Watanabe, Hitoshi Kubosawa, Takeshi Ishii

**Affiliations:** ^1^Department of Orthopaedic Surgery, Chiba Aoba Municipal Hospital, 1273-2 Aoba-cho, Chuo-ku, Chiba 260-0852, Japan; ^2^Department of Pathology, Chiba Aoba Municipal Hospital, 1273-2 Aoba-cho, Chuo-ku, Chiba 260-0852, Japan; ^3^Department of Orthopaedic Surgery, Chiba Cancer Center, 666-2 Nitona-cho, Chuo-ku, Chiba 260-0801, Japan

## Abstract

A 63-year-old male consulted our institution due to worsening of right hip pain for approximately one month. The patient had no apparent functional disorders besides rigidity of the right ankle secondary to childhood poliomyelitis. Plain radiographs demonstrated narrowing of the right hip joint space. Magnetic resonance imaging (MRI) showed unusual findings in the right gluteus medius muscle, suspecting a malignant musculoskeletal tumor. Further examinations clarified acute inflammation caused by* Staphylococcus aureus* with no atypia. After treatment, serum inflammatory markers normalized and MRI showed homogeneous fat signal intensity in the muscle, which was consistent with poliomyelitis. Total hip arthroplasty was performed due to progression of osteoarthritis. Intraoperative findings showed flaccidity of the gluteus medius muscle, and histological examination of the specimen also was compatible with poliomyelitis. Postoperatively there was no hip instability and the patient has been able to resume his previous physical activity. To our knowledge, this is the first report regarding polio survivors combined with septic arthritis, and sole MRI examination was unable to lead to the diagnosis. The current patient demonstrates the possibility that the involved muscles in poliomyelitis exist even in asymptomatic regions, which will be helpful for accurate diagnosis and life guidance in polio survivors.

## 1. Introduction

The use of worldwide vaccine has eradicated poliomyelitis from majority of regions worldwide. Although recently it is even rare to see polio survivors in polio-free regions, poliomyelitis is not a disease of the past. A polio survivor with rigidity of the right ankle consulted us due to right hip pain, in which magnetic resonance imaging (MRI) findings were unusual. Hence we present the MRI findings, pathophysiology, clinical course, and the cautions taken during medical examinations.

## 2. Case Presentation

**Figure 1:**
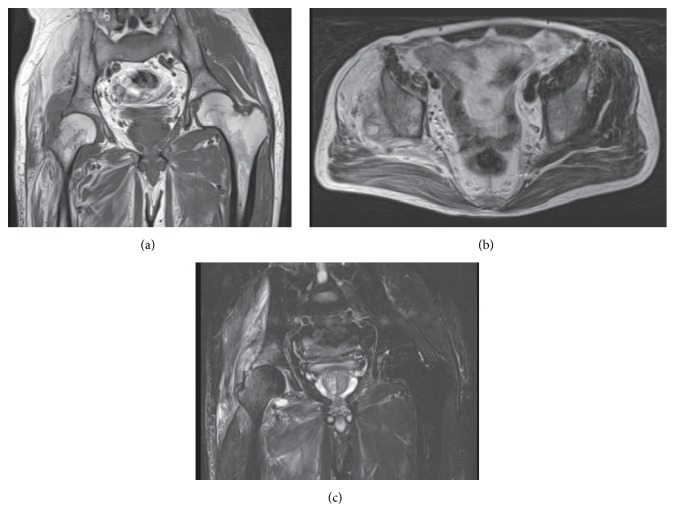
Initial MRI of the hip: (a and b) coronal T1-weighted and axial T2-weighted images showing inhomogeneous fatty signal changes in the gluteus medius muscle and (c) coronal STIR image showing apparent high signal lesions in both the gluteus medius and minimus muscles and slightly high signal lesions in both the femoral head and the acetabular bone with the joint effusion.

A 63-year-old male who had been taking painkillers for his right hip pain for approximately one month consulted our institution due to worsening of pain. The right ankle was almost rigid secondary to childhood poliomyelitis; however, he had no other apparent functional disorders and even played golf as his hobby. On initial examination, the right hip revealed neither marked swelling nor local calor; however, he had difficulty in walking due to severe pain. Plain radiographs demonstrated narrowing of the joint space. MRI showed unusual findings with a mixture of fat and edema in the gluteus medius muscle and slightly bone marrow edema at the adjacent bone with the joint effusion (Figure 1). Hence he was referred to a specialized institution on suspicion of a malignant musculoskeletal tumor.

Serologic tests at the institution showed elevated C-reactive protein at 14.0 mg/dL (0–0.3 mg/dL), although white cell count was normal. An open biopsy revealed apparent purulence from the right hip joint; hence irrigation and debridement were performed. Culture of the purulence revealed* Staphylococcus aureus* and histological findings clarified acute inflammation but no atypia. After two weeks of intravenous antibiotic therapy, oral therapy was initiated and the patient was discharged from the institution to be followed up in our outpatient clinic.

**Figure 2:**
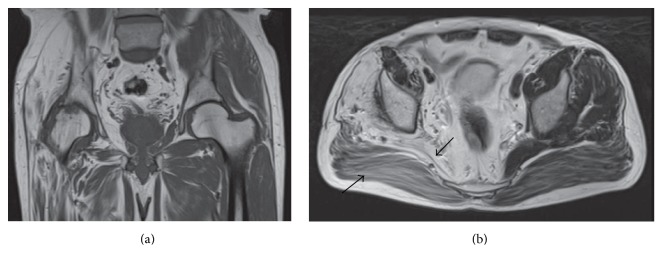
MRI of the hip taken 10 months after the starting of treatment: (a) coronal T1-weighted image showing low signal osteoarthritis change and (a and b) coronal T1-weighted and axial T2-weighted images showing homogeneous fatty signal changes in the gluteus medius muscle and similar appearance even in the other muscles (arrows).

Two months later, inflammatory biomarker levels were back to normal; however plain radiographs revealed progression of osteoarthritis. At ten months after initiation of the treatment, short-inversion-time inversion-recovery (STIR) showed no edematous lesions and no joint effusion; however, T1-weighted image showed low signal osteoarthritis change. Muscle fat signal intensity on both T1- and T2-weighted images was consistent with poliomyelitis (Figure 2). Since the patient was unable to walk due to severe right hip pain, a posterior approach total hip arthroplasty (THA) was planned after one year from normalization of laboratory analysis.

**Figure 3:**
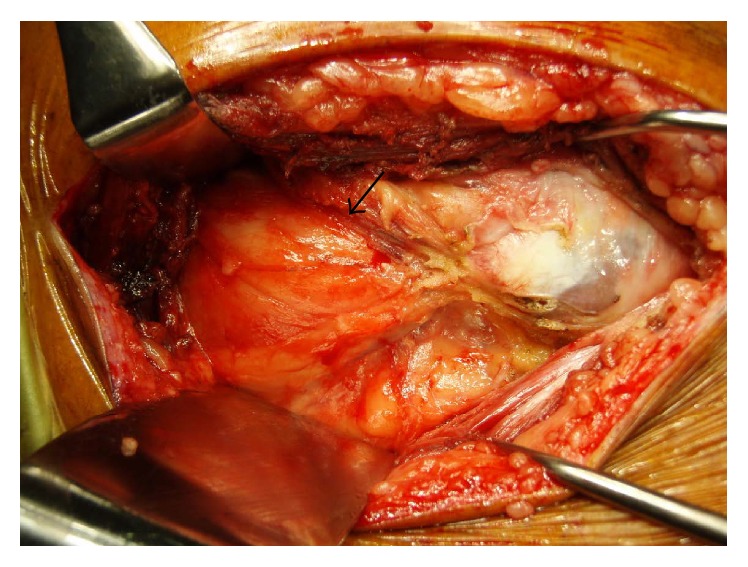
Intraoperative photograph showing yellowish gluteus medius muscle (arrow), which had flaccid tone.

During surgery, a yellowish gluteus medius muscle was confirmed (Figure 3), and several pieces of the muscle were submitted separately for cultures and histological examinations. Resected joint capsule and synovial tissue were also submitted for examinations. Macroscopically apparent purulence was not evident anywhere and bone quality was not poor. Although the gluteus medius muscle was flaccid, hip motion within the ordinary range had no tendency of dislocation; hence the usual prosthesis was implanted with no specific leg length correction (Trident Acetabular Shell and X3, Accolade II; Stryker Orthopaedics, Mahwah, New Jersey, Biolox Delta 32 mm Femoral Head; CeramTec AG, Plochingen, Germany).

**Figure 4:**
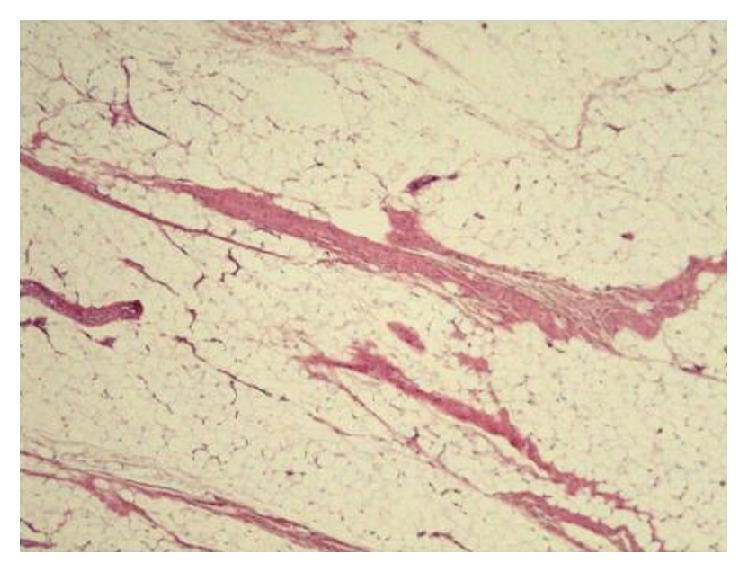
Histological examination of the gluteus medius muscle showing diffuse fat infiltration with a few atrophic muscle fibers, but no acute inflammation and no atypia (H and E, ×40).

All cultures of specimens were negative for bacteria, and all histological findings were negative for acute inflammation and atypia. The specimen from the gluteus medius muscle showed histologically fat infiltration, consistent with the involved muscle in poliomyelitis (Figure 4). Tolerable weight bearing exercises were encouraged from the first postoperative day. Six months after surgery, the right hip muscle strength was grade 5 of 5 for flexors and abductors. During the following 2 years after surgery, the patient had no complaints of pain, besides muscle fatigue, no recurrence of infection, and no prosthetic dislocation. The patient was able to resume his previous physical activities.

## 3. Discussion

Some reports have suggested that the involved muscle in poliomyelitis is characterized by high signal intensity on both T1- and T2-weighted images indicating abnormal fat infiltration [1]. In the current patient, the initial MRI showed fatty signal changes with inhomogeneous edematous lesions in the right gluteus medius muscle (Figure 1). These findings are capable of substantially limiting differential diagnosis such as malignant lymphoma and lipomatous tumors [2–4]. But sole MRI examination was unable to lead to an accurate diagnosis because of no functional disorders secondary to poliomyelitis in the hip joint. Moreover, adult-onset primary septic arthritis is also relatively uncommon, which added to the difficulty in making the diagnosis [5]. In fact, to our knowledge, this is the first report regarding polio survivors combined with septic arthritis. It has been reported that adipose tissue plays a major role in inflammation and the pathogenesis of infectious diseases [6]. The abnormal expansion of inflammation and edematous lesion might be associated with abnormal fatty muscle secondary to poliomyelitis.

Although there have been various postoperative complications in the polio survivors, THA was reported to be a suitable procedure for the painful hip [7, 8]. But the problem in this case was whether infection had been controlled or not. It has been reported that MRI is helpful in assessing the problem; therefore, we made the decision to perform arthroplasty after disappearance of edematous lesions [9]. We believe that postoperative analyses of the retrieval tissues and the patient's clinical course also support quiescent infection.

Polio survivors undergoing THA on their paralytic limb have the risk of prosthetic dislocation and loosening. Fortunately postoperative hip instability in this case was not evident regardless of flaccidity in the gluteus medius muscle, which may be caused by compensatory functions in the remaining motor units. Postpolio syndrome was reported to have a relationship with stress or overuse of remaining motor units, resulting in progressive muscle weakness, abnormal muscle fatigue, and muscle atrophy [10]. In the current patient, even the muscles around the gluteus medius muscle had fat infiltration on MRI, speculating to be not so strong against overwork (Figure 2). The patient actually became conscious of muscle fatigue around the right hip during long distance walking. Based on the MRI findings, the patient was informed of a potential of postpolio syndrome, and we advised avoidance of both overuse and disuse in order not to develop postpolio syndrome [11].

## 4. Conclusion

We presented a polio survivor who underwent THA secondary to osteoarthritis caused by infectious arthritis. This rare case demonstrates the possibility that the involved muscles in poliomyelitis exist even in asymptomatic regions and even the compensatory muscles have fat infiltration. We believe that this knowledge contributes to accurate diagnosis and life guidance in polio survivors.
